# Accidental discovery of cholecystoduodenal fistula during laparoscopic cholecystectomy: a case report

**DOI:** 10.3389/fsurg.2025.1617555

**Published:** 2025-08-13

**Authors:** Rui Huang, Jian-Xing Tian, Xu Deng, Zong-Long Zhu, Wei Tian, Chun-Yuan Yang, Ming Xia, Wei Pan

**Affiliations:** Department of Hepatobiliary and Pancreatic Surgery, The People’s Hospital of Lezhi, Lezhi, China

**Keywords:** gallbladder, duodenum, cholecystoduodenal fistula, gallbladder stones, endovascular fistula

## Abstract

Cholecystoduodenal fistula (CDF) is a rare complication of cholelithiasis. Sometimes, a preoperative examination does not fully detect CDF and may have an impact on the conduct of the surgery. Sometimes, clinicians fail to accurately determine the presence of CDF, which may have an impact on the conduct of the procedure. Here, we report the diagnosis and management of a patient with a CDF that was accidentally detected intraoperatively. The patient was hospitalized twice in our hospital due to excessive inflammation around the gallbladder and combined with choledochal stones. During the second hospitalization, a CDF was accidentally found during laparoscopic cholecystectomy. We repaired the fistula, ligated the cystic duct, and removed the gallbladder laparoscopically while ensuring patient safety. In the absence of preoperative detection of a cholecystoenteric fistula (CEF), intraoperative judgment and postoperative management are of particular importance, which is what will be discussed in this article.

## Introduction

Cholecystoenteric fistula (CEF) is a relatively rare complication of gallbladder stone disease, defined as a spontaneous passage formed between the inflammatory response of the gallbladder and the surrounding adherent gastrointestinal (GI) tract (1). Previous studies have shown that the rate of incidence of CEF in patients with cholelithiasis is 3%–5% and 0.15%–4.8% in all biliary procedures (2, 3). It usually occurs at advanced age and is most often found in the duodenum, which accounts for 75%–80% of all CEFs, followed by the colon and stomach (4, 5). Some cholecystoduodenal fistulas (CDFs) can lead to gallstone intestinal obstruction, most commonly in the first part of the duodenum, known as “Bouveret's syndrome” (6). Advances in diagnostic imaging and endoscopic techniques have greatly improved the accuracy of the preoperative diagnosis of CEF (7). For example, if there is abdominal CT and magnetic resonance cholangiopancreatography (MRCP) suggesting thickening of the gallbladder wall, gallbladder pneumatosis, gallbladder effusion, or air fluid in the gallbladder, the surgeon should have a high suspicion of CEF. But some patients still do not have preoperative clarification of their condition due to the lack of specific clinical signs and symptoms between CEF and simple cholelithiasis. In this article, we highlight the correct intraoperative judgment and strict postoperative management of a patient with a gallbladder-duodenal fistula that was accidentally detected intraoperatively, which ensured the patient's safety while shortening the patient's recovery time as much as possible.

## Case presentation

**Figure 1:**
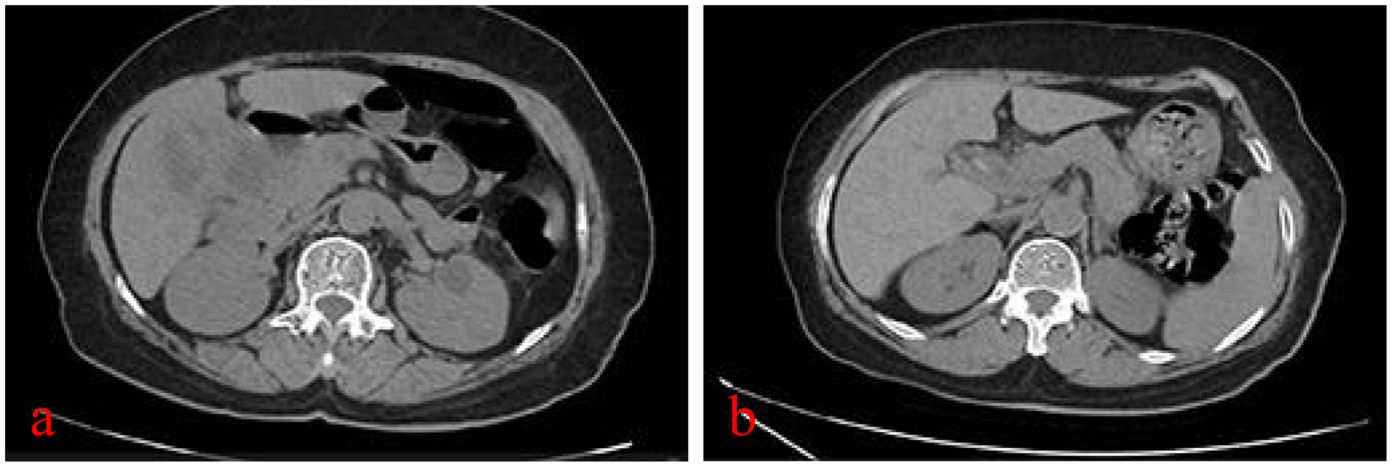
CT plain scan of the abdomen at the time of the patient's two admissions: (**a**) first admission, severe inflammatory reaction in and around the gallbladder; and **(b)** second admission, inflammatory reaction in and around the gallbladder has subsided.

A 77-year-old woman presented to our hospital with “recurrent right upper abdominal pain for 10+ days, aggravated for 10+ h.” The main manifestation is vague pain in the right upper abdomen, with distension and discomfort, accompanied by radiating pain in the back. For over 10 h, the patient's upper abdominal pain had worsened, accompanied by nausea and vomiting of stomach contents. So, she came to our hospital for treatment. The patient had no specific past medical history. Physical examination is characterized by right upper abdominal pressure pain and Murphy (+). Abdominal CT examination suggests structural disorder in the hepatoportal region, unclear display of the gallbladder, and alterations in the corresponding region and adjacent liver parenchyma, accompanied by scattered inflammation in the adjacent peritoneum (Figure 1a). Hematologic findings suggest the following: CA19-9 >1,200 U/ml (reference range 0–43 U/ml); TBil 69.0 μmol/L (reference range ≤23 μmol/L); DBil 44.9 μmol/L (reference range ≤6.8 μmol/L); IBil 24.1 μmol/L (reference range 3.4–23.2 μmol/L); ALT 32 U/L (reference range 7–40 U/L); AST 402 U/L (reference range 13–35 U/L); WBC 13.79 × 10^9^/L (reference range 3.5–9.5 × 10^9^/L); CRP 111.25 mg/L (reference range 0–8 mg/L) (Table 1). Contrast-enhanced CT suggests the following: xanthogranulomatous cholecystitis with limited peritonitis; possible invasion of adjacent liver parenchyma; gastric sinuso-descending colon; and neoplastic lesions cannot be excluded. MRCP suggests multiple gallbladder stones; thickening of the gallbladder wall; poor demarcation of the gallbladder from the adjacent hepatic tissues and intestinal tract. It's considering xanthogranulomatous cholecystitis or gallbladder carcinoma. In addition to this, it suggests a single stone in the pancreatic segment of the common bile duct, with dilatation of the intrahepatic and extrahepatic bile ducts. Combined with the patient's history, signs, and ancillary tests, we came up with the following diagnoses: (1) choledocholithiasis with cholangitis, (2) gallbladder stones with acute cholecystitis, (3) hepatic insufficiency, (4) obstructive jaundice, and (5) gallbladder space-occupying disease. The patient's gallbladder lesion did not exclude the possibility of malignancy and invaded adjacent organs. If the lesion was an inflammatory reaction, it was not suitable for one-stage surgical treatment. If the lesion was a malignant tumor, the opportunity for radical surgery may have been lost. After communicating with the patient and his family, we chose to perform endoscopic retrograde cholangiopancreatography (ERCP) to relieve the biliary obstruction. ERCP suggested the presence of a stone in the common bile duct and a stricture at the lower end of the common bile duct. Therefore, we left a stent in the bile duct. She was recommended that she should be followed up regularly, and the next diagnostic and treatment modality should be decided according to the subsequent changes in the gallbladder lesion.

**Table 1:** Major hematologic test results at the time of the patient's two hospital admissions.

Hematological index	First hospitalization	Second hospitalization
CA19-9 (U/ml)	>1,200	13.79
TBil (μmol/L)	69.0	14.3
DBil (μmol/L)	44.9	4.1
IBil (μmol/L)	24.1	10.2
ALT (U/L)	232	20
AST (U/L)	402	21
WBC (10^9^/L)	13.79	6.21
NEUT (%)	90.40	57.50
NEUT (10^9^/L)	12.47	3.57
CRP (mg/L)	111.25	2.42

Abbreviations: CA19-9, carbohydrate antigen 19-9; TBil, total bilirubin; DBil, direct bilirubin; IBil, indirect bilirubin; ALT, alanine aminotransferase; AST, aspartate aminotransferase; WBC, white blood cell; CRP, C-reactive protein; NEUT, neutrophil.

Six months later, the patient returned to the hospital for further treatment, and abdominal CT suggested that the gallbladder was shrunken, gallbladder stones, with the presence of small air bubble shadows and a slightly thicker gallbladder wall (Figure 1b). Hematologic tests did not indicate abnormality (Table 1). MRCP suggests dilatation of the intrahepatic and extrahepatic bile ducts, possible stones in the middle and upper part of the common bile duct, shrinkage of the gallbladder, and gallbladder stones. Therefore, we performed ERCP again and confirmed the presence of three stones in the common bile duct.

**Figure 2:**
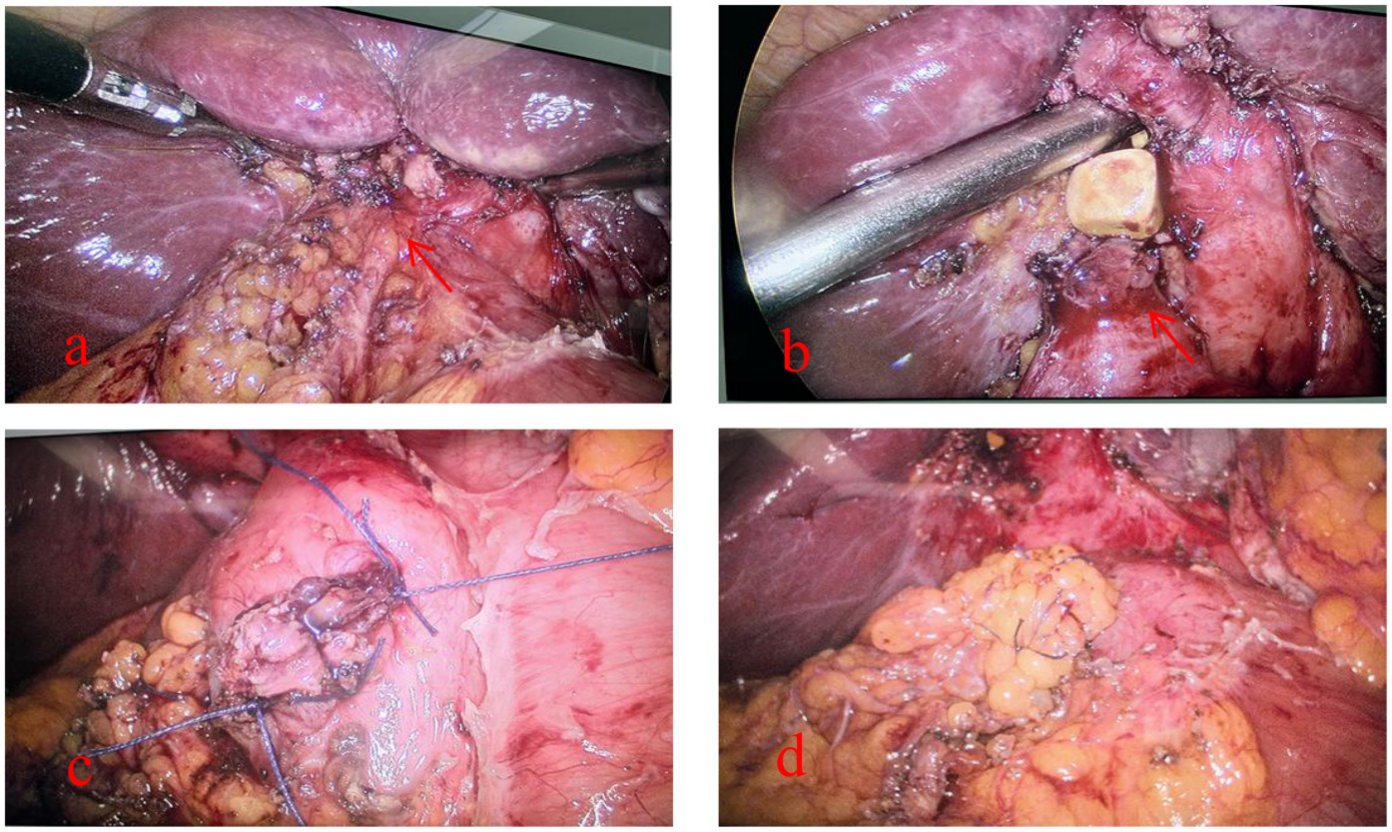
Intraoperative fistula detection and management of the patient: **(a)** tight adhesion of the gallbladder to the duodenum (red arrow), **(b)** accidental discovery of the fistula during separation of the adhesion (red arrow), **(c)** suture of the duodenal fistula, and **(d)** coverage of the duodenal fistula by the greater omentum after the suture has been closed.

Combining the patient's ancillary tests and our experience, we concluded that an inflammatory lesion of the gallbladder was more likely and performed a laparoscopic cholecystectomy (LC). Intraoperatively, we found that the gallbladder was markedly atrophied and adherent to the duodenum, and during the process of separating the adhesions, we found that the duodenum had formed an internal fistula with the gallbladder. The diameter of the fistula was approximately 1 cm. After discussing the patient's condition with the Department of Gastrointestinal Surgery, we decided to adequately clean the scar tissue around the duodenal fistula and close the duodenal fistula with interrupted sutures using 3-0 Vicryl, and a small amount of greater omentum was used to cover the suture and secure it (Figure 2). In addition, we ligated the cystic duct and removed the gallbladder while ensuring that there was no damage to the common bile duct. An abdominal drain was left in the vicinity of the fistula to facilitate postoperative observation of the presence or absence of an enterocutaneous fistula. Postoperatively, the patient was left with a gastric tube for GI decompression, used Omeprazole and Somatostatin to suppress digestive fluid secretion. Of course, total parenteral nutrition is also indispensable. At 6 days postoperatively, a repeat abdominal CT suggested that there was no obvious abnormality in the abdominal cavity, so we removed the gastric tube. We asked the patient to start the dietary regimen and gradually increase the amount. The patient did not report intestinal leakage during this process, so we removed the abdominal drain at 9 days postoperatively, following which the patient recovered and was discharged at 11 days postoperatively. The patient was very cooperative with us during the treatment.

At follow-up, the patient reported no abdominal pain, bloating, or dyspepsia after discharge.

## Discussion

CEF is a condition first described by Thomas Bartholin in 1654 that displaces gallstones into the GI tract and is a rare complication of cholelithiasis (6). In the elderly population, CEF has a relatively high morbidity and mortality associated with it, and despite improvements in imaging techniques, diagnosing CEF remains challenging (7). One study showed that only 31.0% of patients were diagnosed with CEF preoperatively (8). As a result, most patients are unable to adequately assess their condition preoperatively, leading to inadequate preparation, which can impede the performance of the procedure. To further improve preoperative diagnosis rates, some studies have recommended the following: (1) Recurrent cholecystitis over a long period of time (especially >5 years) is a risk factor that should be brought to the attention of the surgeon; (2) Although accurate diagnosis by ultrasound (US) testing may be difficult, signs such as gallbladder wall thickening, gallbladder atrophy, and cholecystitis/gallbladder effusion are important clues to CEF; (3) Abdominal CT (plain or contrast-enhanced scan), especially coronal reconstruction, is essential for effective recognition of signs of poorly defined borders between the gallbladder and the GI tract. In addition, surgeons should be highly suspicious of CEF if there is a combination of gallbladder wall thickening, gallbladder pneumatosis, gallbladder effusion, or air fluid in the gallbladder; (4) MRCP has similar signs to CT scan. Notably, MRCP is more helpful when CEF is combined with choledocholithiasis or Mirizzi syndrome; (5) Upper GI imaging and gastroscopy/colonoscopy should be considered when CEF is suspected to look for fistulae or communication between the GI tract and the gallbladder; (6) In patients presenting with intestinal obstruction, the typical signs, also known as Rigler's triad, include dilatation of the intestinal trocars, calcified margins, or total calcified gallstone impaction, which, when combined with the above signs, make diagnosis relatively easy (9). In addition, Yamashita et al. (10) reported that ERCP is the most valuable diagnostic method for revealing the presence or absence of CEF. However, ERCP is an invasive method limited to patients with jaundice and/or choledocholithiasis, and ERCP does not always find incomplete fistulas (11). In another study, CEF was diagnosed in only four of seven patients with CEF who underwent ERCP for choledocholithiasis (8). In summary, there are some difficulties in diagnosing CEF preoperatively. Preoperative evaluation of CEF may be helpful by analyzing the characteristics of the patient's history and combining diverse auxiliary examinations.

However, failure to diagnose CEF preoperatively may pose a challenge to surgeons, who may need to perform unexpectedly complex and lengthy procedures (5). For example, if CEF is not identified, surgeons may accidentally tear the infected GI tract, causing contamination of the peritoneum with intestinal contents (12). Previous studies have recommended that CEF should be treated with open cholecystectomy with resection and suturing of the fistula (4). However, there is now a growing number of reports showing that with more experience and improved techniques, more and more cases are reported to have been successfully treated for CEF using a laparoscopic approach with all the benefits of minimally invasive surgery (12–15). At the same time, because laparoscopic treatment of CEF is complex and dangerous, studies have suggested the following: (1) Ensure that each step of the operation is meticulously performed under direct vision. (2) The surgeon must be experienced in the techniques of advanced laparoscopic surgery, including laparoscopic suturing. (3) If the sinus between the gallbladder and the GI tract cannot be completely exposed, some amount of gallbladder tissue can be retained when repairing the sinus. (4) If the anatomical structure of the gallbladder triangle is not clear, partial cholecystectomy is a safe and effective surgical procedure (7). Fortunately, we fully exposed the fistula between the gallbladder and the duodenum, dissected the gallbladder triangle, and completed the repair of the fistula and cholecystectomy through laparoscopy.

## Conclusion

In this case, during the patient's first hospitalization, neither abdominal CT nor MRCP suggested gallbladder pneumoperitoneum; at the second hospitalization, although MRCP did not suggest gallbladder pneumoperitoneum, abdominal CT had suggested gallbladder pneumoperitoneum. We should be alert to the presence of CDF and make adequate preoperative preparations. In addition, during LC, surgeons should be highly suspicious of CEF if they find extensive inflammatory adhesions around the gallbladder in close relation to the GI tract. Fortunately, after the discovery of CEF, we analyzed the adequacy of the patient's preoperative preparation, did our best to improve the deficiencies, and completed the surgical treatment by laparoscopy. Patients recover quickly and without complications after surgery, which is inextricably linked to the surgeon's extensive experience, surgical skills, and strict postoperative management. Unfortunately, on the patient's second hospitalization, abdominal CT suggested gallbladder pneumoperitoneum, and during the ensuing ERCP operation, we did not deliberate efforts to look for the presence of CDF.

## Data Availability

The raw data supporting the conclusions of this article will be made available by the authors without undue reservation.
